# Novel Web Service Selection Model Based on Discrete Group Search

**DOI:** 10.1155/2014/460593

**Published:** 2014-04-15

**Authors:** Jie Zhai, Zhiqing Shao, Yi Guo, Haiteng Zhang

**Affiliations:** Department of Computer Science and Engineering, East China University of Science and Technology, Shanghai 200237, China

## Abstract

In our earlier work, we present a novel formal method for the semiautomatic verification of specifications and for describing web service composition components by using abstract concepts. After verification, the instantiations of components were selected to satisfy the complex service performance constraints. However, selecting an optimal instantiation, which comprises different candidate services for each generic service, from a large number of instantiations is difficult. Therefore, we present a new evolutionary approach on the basis of the discrete group search service (D-GSS) model. With regard to obtaining the optimal multiconstraint instantiation of the complex component, the D-GSS model has competitive performance compared with other service selection models in terms of accuracy, efficiency, and ability to solve high-dimensional service composition component problems. We propose the cost function and the discrete group search optimizer (D-GSO) algorithm and study the convergence of the D-GSS model through verification and test cases.

## 1. Introduction

We have proposed a novel approach for the verification of service composition with contracts [1]. The approach properties of the generic specification [2] in Tecton [3] are verified by the Violet [4] system. After verification, a global optimum is selected from a number of instantiations of web service composition components with multiple QoS constraints. Compared with other algorithms that evaluate all feasible composition instantiations (e.g., integer programming [5]), evolutionary algorithms (EAs) (e.g., genetic algorithm [6]), which are nature-inspired optimization algorithms, are simple and flexible. Given their characteristics, EAs have been used to solve the service selection problem. We proposed a novel optimization model named discrete group search service (D-GSS) that mainly employs the group search optimizer (GSO) algorithm [7]. The D-GSS model has competitive performance compared with other EAs in terms of accuracy, convergence speed, and ability to solve high-dimensional multimodal problems. On the basis that GSO can solve continuous optimization problems and that service selection can solve discrete instantiations, we present an evolutionary algorithm called discrete group search optimizer (D-GSO) to select the best instantiation that has the lowest cost evaluated by the cost function. The cost function consists of the utility function and the weight for every QoS attribute. We also verify and simulate results to analyze the convergence of the D-GSS model.

The rest of the paper is organized as follows.  Section 2 describes the D-GSS model.  Section 3 presents a detailed introduction of the cost function, and Section 4 discusses the D-GSO algorithm and applies the algorithm for the problem on searching for the global optimum from discrete instantiations.  Section 5 introduces the convergence analysis of the D-GSS model. Finally, Section 6 concludes the paper.

## 2. Distribute Group Search Optimizer

In this paper, we present a novel algorithm named D-GSS toward the atomic service selection of composing complex services with multiple QoS constraints. The population of the D-GSO algorithm is called a group searching for unknown optima in the services composition problem and each individual in the population is called a member.

In the *n*-dimensional search space *I* about composition component, every dimension represents a class of generic service denoted as *I*_*i*_. The *i*th member *X*_*i*_ in the space *I* is denoted as follows:

(1) $$\begin{array}{c} I=\left\{I_{1},I_{2},\ldots ,I_{n}\right\},\\ {\;X}_{i}=\left\{x_{i}^{1},x_{i}^{2},\ldots ,x_{i}^{n}\right\}, \end{array}$$

where *x*_*i*_^*j*^ ∈ *I*_*j*_. The *i*th member *X*_*i*_  at the *k*th iteration has a current position *X*_*i*_^*k*^ ∈ *R* ^*n*^ and *X*_*i*_^*k*^ is corresponding to an instantiation of services composition component.

A head angle *ϕ*_*i*_^*k*^ is the position of the member; *ϕ*_*i*_^*k*^ = (*ϕ*_*i*_1__^*k*^, *ϕ*_*i*_2__^*k*^,…, *ϕ*_*i*_(*n*−1)__^*k*^) ∈ *R* ^*n*−1^. The search direction of the *i*th member, which is a unit vector *D*_*i*_^*k*^(*ϕ*_*i*_^*k*^) = (*d*_*i*_1__^*k*^, *d*_*i*_2__^*k*^,…, *d*_*i*_*n*__^*k*^) ∈ *R* ^*n*^ that can be calculated from *ϕ*_*i*_^*k*^ via a polar to Cartesian coordinate transformation [7]:

(2) di1k=∏q=1n−1cos⁡⁡(ϕiqk),dijk=sin⁡(ϕi(j−1)k)·∏q=1n−1cos⁡⁡(ϕiqk) (j=2,…,n−1),dink=sin⁡(ϕi(n−1)k).

In D-GSO based on GSO [7] inspired by animal behavior and animal searching behavior, a group consists of three types of members: only one producer is assumed to have the lowest cost at each searching bout, and the remaining members are assumed to be scroungers and dispersed members. At each iteration, a group member representing the most promising instantiation and conferring the lowest fitness value is chosen as the producer. It then stops and scans the environment to seek optimal instantiation. The scanning field is characterized by maximum pursuit angle *θ*_max⁡_ and maximum pursuit distance *l*_max⁡_. The apex is the position of the producer. All scroungers will join the resource found by the producer according to area copying strategy. The rest of the group members will be dispersed from their current positions for randomly distributed better instantiations. To handle the bounded search space, the following strategy is employed: when a member is outside the search space, the member will return into the search space by setting the variables that violated the bounds into their previous values.

**Figure 1:**
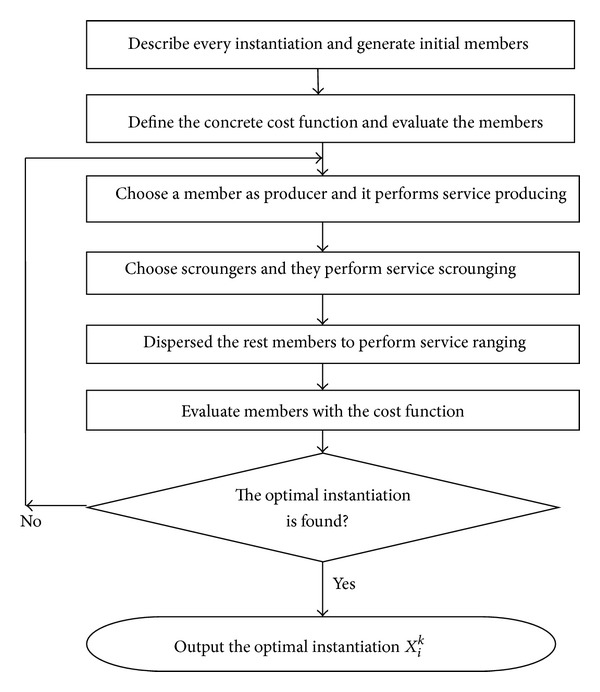
Flowchart of the D-GSS model.

The details of D-GSO (see Figure 1) are introduced as follows.

- Suppose that *n* classes of generic services exist in the *n*-dimensional composition component; each class has *N*_*i*_  (1 ≤ *i* ≤ *n*) candidate services in a special sequence.
- Define the concrete cost function of the specific composition component. The cost function is defined by the QoS attributes of the component services as well as their integration relationships, such as sequential, parallel, conditional, or loop. Generate initial members from all instantiations and evaluate the members according to the cost function.
- Choose a member with the lowest cost as producer. The producer produces on the basis of the discrete GSO algorithm.
- Randomly select 80% of the remaining members to perform scrounging.
- The remaining members will be dispersed from their current instantiations to perform ranging.
- Evaluate all members according to the cost function. If no optimal instantiation with multiple QoS constraints is found, reallocate the role of every member on the value of the cost.

## 3. Cost Function

A “generic service” is a collection of atomic web services with a common functionality, but different nonfunctional properties (e.g., time and quality). Each atomic service may provide a series of QoS parameters, such as service time, cost, reliability, and availability. Users can set the number of QoS values to be considered and can set the weights of the QoS values according to their requirements. In our study, each user has *k* QoS attribute constraints in their QoS requirements: *Q*_*c*_ = [*Q* ^1^,…, *Q* ^*k*^]. We focus on the QoS service selection problem, in which multiple QoS constraints must be satisfied. We present the cost function to help in the selection of the best services. The following steps are involved in the creation of the cost function.

- Each QoS attribute must be quantitative. Service functionalities can be evaluated by several QoS properties. Some QoS attributes, for example, security and reliability, are difficult to measure quantitatively. For these criteria, we employ the linguistic expression set *L*1 = {VP, MP, P, M, G, MG, VG}, where VP is very poor, MP is medium poor, P is poor, M is medium, G is good, MG is medium good, and VG is very good. When calculating the cost function, set *L*1 is transformed into the corresponding quantitative set *P*1 = {0.15,0.3,0.45,0.6,0.75,0.9,1}.
- Global QoS attributes (*q*_*c*_ = [*q* ^1^,…, *q* ^*k*^]) are needed to describe the performance of an instantiation of service composition component. Every global QoS attribute is aggregated by the QoS attributes of all atomic services considering the integration relationships of the global QoS attribute. Each service has four main basic structures: (1) the sequential structure, which represents *n* services that are invoked one by one; (2) the loop structure, which represents one service that is repeated *p* times; (3) the conditional structure, which represents only one branch that is selected to be invoked from *n* branches; (4) the parallel structure, which represents *n* branches that are invoked simultaneously. The complete structure of the service composition component consists of the above four basis structures. Every global QoS attribute has its own aggregated method. We sort the QoS aggregated methods into three types: (1) the summation method (e.g., cost), in which the fees must be accumulated by the user to pay for invoking the services; (2) the continued multiplication method (e.g., availability), in which global availability can be computed as the product of the ratios of all atomic service availability; (3) the average method (e.g., reputation), in which global reputation is the average value of the related service reputation. We present all particulars (see Table 1) of these three methods with sequential, parallel, conditional, or loop structures. In Table 1, *c* ^*i*^ is a 0-1 variable. If condition *c* ^*i*^ is satisfied, then we define *c* ^*i*^ = 1; otherwise, *c* ^*i*^ = 0.
- After the values of [*q* ^1^,…, *q* ^*k*^] and [*Q* ^1^,…, *Q* ^*k*^] are evaluated, we present a utility function to describe the relationship between *q* ^*i*^ and *Q* ^*i*^. Two types of QoS criteria are available, that is, cost and benefit. In the cost criterion, variables (e.g., response time) with higher values have lower qualities. In the benefit criterion, variables (e.g., availability) with higher values have higher qualities. The utility function synthesizes the cost and benefit criteria.

**Definition 1 (utility function)**

Suppose that a global QoS attribute *q* ^*i*^  (1 ≤ *i* ≤ *k*) and its constraint *Q* ^*i*^ of an instantiation *S* ^*j*^ exist, the utility function is defined as follows:

(3) $$\begin{array}{c} U\left(q_{i}^{j},Q_{i}^{j}\right)=\{\begin{array}{cc} \frac{q_{i}^{j}}{Q_{i}^{j}}, & \text{if}\;\;q_{i}^{j}\;\;\text{is}\;\text{the}\;\;\text{cost}\;\;\text{criterion},\\ 2-\frac{q_{i}^{j}}{Q_{i}^{j}}, & \text{if}\;\;q_{i}^{j}\;\;\text{is}\;\text{the}\;\;\text{benefit}\;\;\text{criterion}. \end{array} \end{array}$$

If the global QoS attribute *q* ^*i*^ satisfies the requirement of the QoS constraint *Q* ^*i*^, then *U*(*q*_*i*_^*j*^, *Q*_*i*_^*j*^) ≤ 1; otherwise *U*(*q*_*i*_^*j*^, *Q*_*i*_^*j*^) > 1.

(iv) The cost function is based on the values of the utility function and the weights the user defined. The better the instantiation is, the lower the quality of the cost function result becomes.

**Definition 2 (cost function)**

Suppose that an instantiation *S* ^*j*^ exists in the QoS attributes *q*_*c*_ = [*q*_1_^*j*^,…, *q*_*k*_^*j*^], QoS constraints *Q*_*c*_ = [*Q*_1_^*j*^,…, *Q*_*k*_^*j*^], and the weights for each QoS attribute; then the cost function is defined as follows:

(4) $$\begin{array}{c} F\left(X_{j},q_{c},Q_{c}\right)=\overset{k}{\underset{i=1}{\sum }}w_{i}^{j}U\left(q_{i}^{j},Q_{i}^{j}\right), \end{array}$$

where ∑_*i*=1_^*k*^ *w*_*i*_^*j*^ = 1 and *U*(*q*_*i*_^*j*^, *Q*_*i*_^*j*^) ≤ 1  (1 ≤ *i* ≤ *k*).

The objective of this paper is to employ D-GSO to get the optimal solution of the following model:

(5) $$\begin{array}{c} \min\left(F\left(X_{j}\right)\right)=\overset{k}{\underset{i=1}{\sum }}w_{i}^{j}U\left(q_{i}^{j},Q_{i}^{j}\right), \end{array}$$

where *X*_*j*_ ∈ *R* ^*n*^.

## 4. D-GSO Algorithm

The GSO algorithm [7] designs optimum searching strategies to solve continuous optimization problems. However, service selection is a discrete problem. Therefore, we present an evolutionary algorithm named D-GSO that can handle composition components with discrete atomic services. The steps of the D-GSO algorithm are described in Algorithm 1. In the D-GSO algorithm, round(*x*) represents a round function for half adjust result. Suppose that sub*X*_*i*_^*h*^. represents [1_*i*_^*h*^, 2_*i*_^*h*^,…, *n*_*i*_^*h*^], which are the subscripts of atomic services composing an instantiation *X*_*i*_^*h*^ about the *i*th member *X*_*i*_ at the *h*th iteration. At the (*h* + 1) iteration, the transformation of the subscripts by the following formulas is [1_*i*_^*h*+1^, 2_*i*_^*h*+1^,…, *n*_*i*_^*h*+1^] relating to a new instantiation (see Algorithm 1).

## 5. Convergence Analysis of the D-GSS Model

### 5.1. Convergence Verification

In this section, we verified the convergence of the D-GSS model. After *n* iterations, the best instantiation with the lowest cost can be determined with the cooperation of the producer and some scroungers and rangers.

**Lemma 3**

If *X* represents the space of all instantiations *X*_*i*_^*k*^ and *P* represents the space of the producer, then *X* = *P*.

**Proof**

(1)  *l*_max⁡_ denotes the maximum distance between two points in space *X*. By using (3) to (6), we can equate space *P* to a sphere that has center *X*_*h*_^*p*^ possessing sub(*X*_*h*_^*p*^) and radius *l*_max⁡_. Thus, *X* ⊂ *P*.

(2) The following strategy is employed by using the D-GSS model: when a member in space *P* is outside space *X*, the member will return into space *X* by setting the variables that violated the bounds to their previous values. Therefore, *P* ⊂ *X*.

(3) Thus, we conclude that *X* = *P*.

**Theorem 4**

The costs of instantiations in the group will converge to the global optimum that corresponds to the best instantiation with the lowest cost.

**Proof**

In the D-GSS model at the *h*th iteration,

(1) the producer *S* ^*p*^ behaves according to (ii)–(iv) in Algorithm 1. By applying the D-GSO algorithm, we can derive the following:
  (6) cost(Xh+1p) =min⁡(cost(Xhp),cost(Xz),cost(Xr),cost(Xl)),
(2) the scroungers *X*_*h*+1_^*s*^ will approach the producer through (vii) in Algorithm 1,
(3) the rangers *X*_*h*+1_^*r*^ will disperse from a group to perform random walks via (viii) and (ix) in Algorithm 1 to avoid entrapments in the local minima,
(4) finally, we calculate the costs of all instantiations in the group and reallocate their roles. The cost of the new producer is shown as follows:
  (7) $$\begin{array}{c} \text{cost}\left(X_{h+1}^{p}\right)=\min\left(\text{cost}\left(X_{h+1}^{p}\right),\text{cost}\left(X_{h+1}^{s}\right),\text{cost}\left(X_{h+1}^{r}\right)\right). \end{array}$$

We conclude that cost(*X*_*h*+1_^*p*^) ≤ cost(*X*_*h*_^*p*^) by using (6) and (7), which means that the cost of the producer is monotonically decreasing. A global optimum, which has the lowest cost in all instantiations, exists. As stated in the proof of Lemma 3, *X* = *P*. Therefore, the infimum of cost(*X* ^*p*^) is cost (global optimum); that is, after *n* iterations, the instantiation *X* ^*p*^ converges to the global optimum.

### 5.2. Simulation Convergence Results

**Figure 2:**
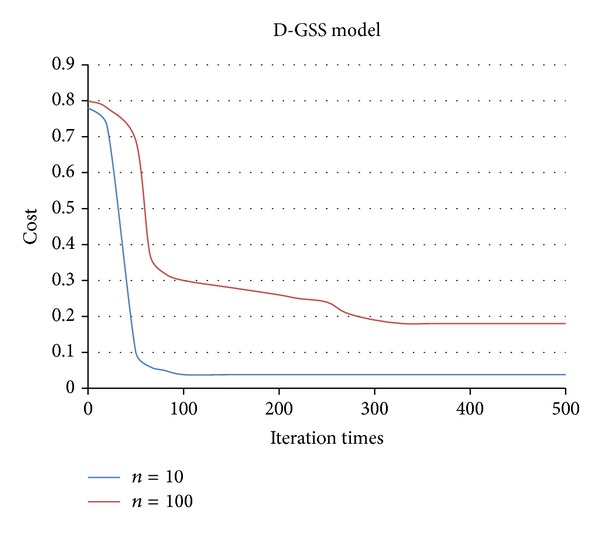
Convergence for *n* = 10,100.

The parameter setting of the D-GSS model is summarized as follows. *M* classes of generic services are present in the complex composition component, in which each class has 50 candidate services that has 10 QoS attributes. The service requestor provides 10 QoS attribute constraints as well as the weights for each QoS attribute. Overall, 51 initial instantiations *X* ^*i*^ with *U*(*q*_*t*_^*i*^, *Q*_*t*_^*i*^) ≤ 1  (1 ≤ *t* ≤ 10) are selected at random in all instantiations. The initial head angle *ϕ* ^0^ of each individual is set to (*π*/4,…, *π*/4). The constant *a* is given by $\text{round}(\sqrt{n+1})$. The maximum pursuit angle *θ*_max⁡_ is *π*/*a* ^2^. The maximum turning angle *α*_max⁡_ is set to *θ*_max⁡_/2. Suppose *n* = 10,100; the relations between the cost of the producer and the iteration times within 500 runs are shown in Figure 2. The experimental results show that the cost of the producer always converges to the optimum of the low- or high-dimensional service composition component. The experiments were conducted on a PC with 2.50 GHz Intel Processor and 8.0 GB RAM. All programs were written and executed in Java. The operating system was Microsoft Windows 7.

## 6. Conclusion

In this paper, we describe a new evolutionary approach for multiconstraints service selection on the basis of the D-GSS model. We propose the cost function and the D-GSO algorithm for searching the global optimum from discrete instantiations of the service composition component. The convergence of the D-GSS model is verified via several formal proofs and simulations. This model has an outstanding advantage in terms of solving high-dimensional service composition problems. In the future, we hope to search for the global optimum under a dynamic heterogeneous environment by using the D-GSS model.

## Figures and Tables

**Algorithm 1:**
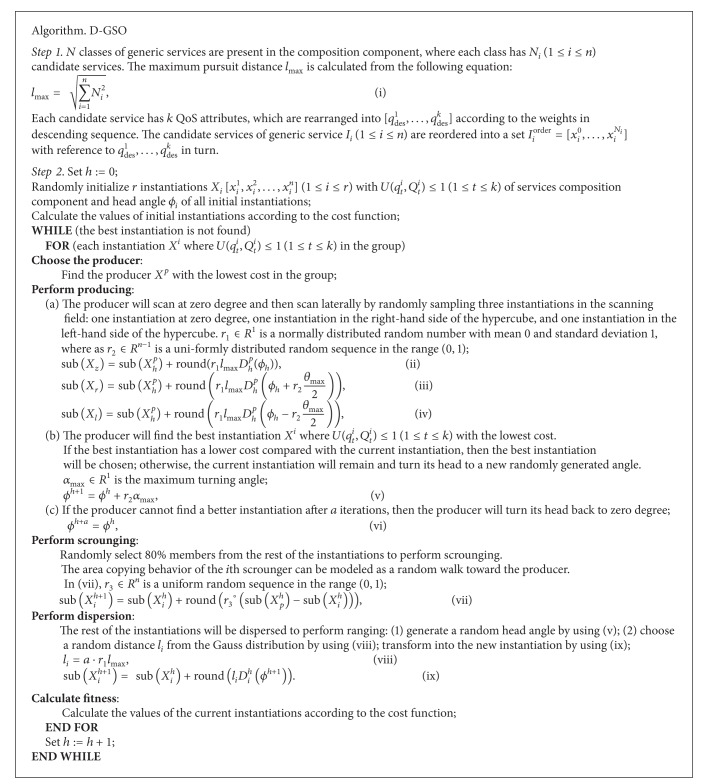
Procedure for the D-GSO algorithm.

**Table 1:** Aggregated methods for global QoS attributes.

Method	Sequential	Loop	Choice	Parallel
Summation	$\overset{n}{\underset{i=1}{\sum }}q^{i}$	*pq* ^*i*^	$\overset{n}{\underset{i=1}{\sum }}c^{i}q^{i}$	$\overset{n}{\underset{i=1}{\sum }}q^{i}$

Continued multiplication	$\prod_{i=1}^{n}q^{i}$	*q* ^*i*^	$\overset{n}{\underset{i=1}{\sum }}c^{i}q^{i}$	$\prod_{i=1}^{n}q^{i}$

Average	$\frac{1}{n}\overset{n}{\underset{i=1}{\sum }}q^{i}$	*q* ^*i*^	$\overset{n}{\underset{i=1}{\sum }}c^{i}q^{i}$	$\frac{1}{n}\overset{n}{\underset{i=1}{\sum }}q^{i}$
